# Supplementary material for: Integrative taxonomy reveals three new species and one new record of Psychropotes (Holothuroidea, Elasipodida, Psychropotidae) from the Kermadec Trench region and the Wallaby-Zenith Fracture Zone
Source: PeerJ. 2025 Feb 21;13:e18806. doi: 10.7717/peerj.18806 (PMC11849507; doi:10.7717/peerj.18806)
Supplement: Supplemental Information 2 [file peerj-13-18806-s002.docx]

**Supplementary Table S2. GenBank accession numbers, localities, voucher information, and source of all species and sequences used in this study.**

| **Species** | **Locality** | **Voucher number** | **GenBank accession number** | | **Sources** |
| --- | --- | --- | --- | --- | --- |
|  |  |  | **16S** | **COI** |  |
| *Psychropotes verrucicaudatus* | South China Sea | MBM286092 | MH077589 | MH077588 | *Xiao et al.*, *2019* |
| *Psychropotes verrucicaudatus* | Clarion-Clipperton Zone | CCZ_086 | N/A | ON400703 | *Bribiesca-Contreras et al.*, *2022* |
| *Psychropotes dyscrita* | Clarion-Clipperton Zone | CCZ_083 | N/A | ON400702 | *Bribiesca-Contreras et al.*, *2022* |
| *Psychropotes moskalevi* | Northwest Pacific Ocean | SO223-5-11-H7 | MN310401 | MN313653 | *Gebruk*, *Kremenetskaia & Rouse*, *2020* |
| *Psychropotes moskalevi* | Northwest Pacific Ocean | SO223-10-11-H17 | MN310400 | MN313650 | *Gebruk*, *Kremenetskaia & Rouse*, *2020* |
| *Psychropotes moskalevi* | Northwest Pacific Ocean | KB-73 | N/A | MN313651 | *Gebruk*, *Kremenetskaia & Rouse*, *2020* |
| *Psychropotes moskalevi* | Northwest Pacific Ocean | KB-1132 | N/A | MN313652 | *Gebruk*, *Kremenetskaia & Rouse*, *2020* |
| *Psychropotes moskalevi* | Northwest Pacific Ocean | KB-1227 | N/A | MN313654 | *Gebruk*, *Kremenetskaia & Rouse*, *2020* |
| *Psychropotes moskalevi* | Northwest Pacific Ocean | KB-1486 | N/A | MN313655 | *Gebruk*, *Kremenetskaia & Rouse*, *2020* |
| *Psychropotes raripes* | Northwest Pacific Ocean | SB-4-3 | MN310403 | MN313656 | *Gebruk*, *Kremenetskaia & Rouse*, *2020* |
| *Psychropotes pawsoni* | East Pacific | ECH00036 | KU987543 | KU987474 | *Gubili et al*., *2017* |
| *Psychropotes longicauda* | Clarion-Clipperton Zone | SIO-BIC E4074 | KX856724 | N/A | *Miller et al*., *2017* |
| *Psychropotes longicauda* | Mexico: Pescadero Basin, Gulf of California, California | SIO-BIC E6788 | N/A | OR082747 | *Mongiardino Koch et al*., *2023* |
| *Psychropotes longicauda* | Indian Ocean | NMV-F308333 | PP817910 | N/A | *Mackenzie et al*., *2024* |
| *Psychropotes* sp. 1 | Pacific | Unavailable data | N/A | KU987469 | *Gubili et al*., *2017*; *Gebruk*, *Kremenetskaia & Rouse*, *2020* |
| *Psychropotes* sp. 2 | Pacific | Unavailable data | N/A | KU987470 | *Gubili et al*., *2017*; *Gebruk*, *Kremenetskaia & Rouse*, *2020* |
| *Psychropotes* sp. 3 | Northeast Atlantic | Unavailable data | N/A | KU987471 | *Gubili et al*., *2017*; *Gebruk*, *Kremenetskaia & Rouse*, *2020* |
| *Psychropotes* sp. 4 | Pacific | Unavailable data | N/A | KU987472 | *Gubili et al*., *2017*; *Gebruk, Kremenetskaia & Rouse*, *2020* |
| *Psychropotes* sp. 5 | Northeast Atlantic | Unavailable data | N/A | KU987473 | *Gubili et al*., *2017*; *Gebruk*, *Kremenetskaia & Rouse*, *2020* |
| *Psychropotes* sp. 6 | Pacific | Unavailable data | N/A | KU987474 | *Gubili et al*., *2017*; *Gebruk*, *Kremenetskaia & Rouse*, *2020* |
| *Psychropotes* sp. 7–sp.10 | Southern Indian | Unavailable data | N/A | KU987475-987478 | *Gubili et al*., *2017*; *Gebruk*, *Kremenetskaia & Rouse*, *2020* |
| *Psychropotes* sp. 11 | Antarctic | Unavailable data | N/A | KU987479 | *Gubili et al*., *2017*; *Gebruk*, *Kremenetskaia & Rouse*, *2020* |
| *Psychropotes* sp. 12 | Northeast Atlantic | Unavailable data | N/A | KU987480 | *Gubili et al*., *2017*; *Gebruk*, *Kremenetskaia & Rouse*, *2020* |
| *Psychropotes* sp. 13 | Antarctic | Unavailable data | N/A | KU987481 | *Gubili et al*., *2017*; *Gebruk*, *Kremenetskaia & Rouse*, *2020* |
| *Psychropotes* sp. 14 | Pacific | Unavailable data | N/A | KU987482 | *Gubili et al*., *2017*; *Gebruk*, *Kremenetskaia & Rouse*, *2020* |
| *Psychropotes* sp. 15 | Northeast Atlantic | Unavailable data | N/A | KU987483 | *Gubili et al*., *2017*; *Gebruk*, *Kremenetskaia & Rouse*, *2020* |
| *Psychropotes* sp. 16 | Pacific | Unavailable data | N/A | KU987484 | *Gubili et al*., *2017*; *Gebruk*, *Kremenetskaia & Rouse*, *2020* |
| *Psychropotes* sp. 17 | Northeast Atlantic | Unavailable data | N/A | KU987485 | *Gubili et al*., *2017*; *Gebruk*, *Kremenetskaia & Rouse*, *2020* |
| *Psychropotes* sp. 18 | Pacific | Unavailable data | N/A | KU987486 | *Gubili et al*., *2017*; *Gebruk*, *Kremenetskaia & Rouse*, *2020* |
| *Psychropotes* sp. 19 | Pacific | Unavailable data | N/A | KU987487 | *Gubili et al*., *2017*; *Gebruk*, *Kremenetskaia & Rouse*, *2020* |
| *Psychropotes* sp. 20 | Northeast Atlantic | Unavailable data | N/A | KU987488 | *Gubili et al*., *2017*; *Gebruk*, *Kremenetskaia & Rouse*, *2020* |
| *Psychropotes* sp. 21 | Pacific | Unavailable data | N/A | KU987489 | *Gubili et al*., *2017*; *Gebruk*, *Kremenetskaia & Rouse*, *2020* |
| *Psychropotes* sp. 22 | Northeast Atlantic | Unavailable data | N/A | KU987490 | *Gubili et al*., *2017*; *Gebruk*, *Kremenetskaia & Rouse*, *2020* |
| *Psychropotes* sp. 23 | Northeast Atlantic | Unavailable data | N/A | KU987491 | *Gubili et al*., *2017*; *Gebruk*, *Kremenetskaia & Rouse*, *2020* |
| *Psychropotes* sp. 24 | Northeast Atlantic | Unavailable data | N/A | KU987492 | *Gubili et al*., *2017*; *Gebruk*, *Kremenetskaia & Rouse*, *2020* |
| *Psychropotes* sp. 25 | Northeast Atlantic | Unavailable data | N/A | KU987493 | *Gubili et al*., *2017*; *Gebruk*, *Kremenetskaia & Rouse*, *2020* |
| *Psychropotes* sp. 26 | Northeast Atlantic | Unavailable data | N/A | KU987494 | *Gubili et al*., *2017*; *Gebruk*, *Kremenetskaia & Rouse*, *2020* |
| *Psychropotes* sp. 27 | Northeast Atlantic | Unavailable data | N/A | KU987495 | *Gubili et al*., *2017*; *Gebruk*, *Kremenetskaia & Rouse*, *2020* |
| *Psychropotes* sp. 28 | Northeast Atlantic | Unavailable data | N/A | KU987496 | *Gubili et al*., *2017*; *Gebruk*, *Kremenetskaia & Rouse*, *2020* |
| *Psychropotes* sp. 29 | Pacific | Unavailable data | N/A | KU987497 | *Gubili et al*., *2017*; *Gebruk*, *Kremenetskaia & Rouse*, *2020* |
| *Psychropotes* sp. 30–sp. 52 | Northeast Atlantic | Unavailable data | N/A | KU987498–987520 | *Gubili et al*., *2017*; *Gebruk*, *Kremenetskaia & Rouse*, *2020* |
| *Psychropotes* sp. 53 | Northeast Atlantic | Unavailable data | N/A | KU987521 | *Gubili et al*., *2017*; *Gebruk*, *Kremenetskaia & Rouse*, *2020* |
| *Psychropotes* sp. 54–sp. 57 | Northeast Atlantic | Unavailable data | N/A | KU987522–987525 | *Gubili et al*., *2017*; *Gebruk*, *Kremenetskaia & Rouse*, *2020* |
| *Psychropotes* sp. 58 | Northeast Atlantic | Unavailable data | N/A | KU987526 | *Gubili et al*., *2017*; *Gebruk*, *Kremenetskaia & Rouse*, *2020* |
| *Psychropotes* sp. 59 | Northeast Atlantic | Unavailable data | N/A | 987527 | *Gubili et al*., *2017*; *Gebruk*, *Kremenetskaia & Rouse*, *2020* |
| *Psychropotes* sp. 60 | Northeast Atlantic | Unavailable data | N/A | KU987528–987530 | *Gubili et al*., *2017*; *Gebruk*, *Kremenetskaia & Rouse*, *2020* |
| *Psychropotes* sp. 61 | Northeast Atlantic | Unavailable data | N/A | KU987529 | *Gubili et al*., *2017*; *Gebruk*, *Kremenetskaia & Rouse*, *2020* |
| *Psychropotes* sp. 62 | Northeast Atlantic | Unavailable data | N/A | KU987530 | *Gubili et al*., *2017*; *Gebruk*, *Kremenetskaia & Rouse*, *2020* |
| *Psychropotes* sp. 63–sp. 67 | Northeast Atlantic | Unavailable data | N/A | KU987531–987535 | *Gubili et al*., *2017*; *Gebruk*, *Kremenetskaia & Rouse*, *2020* |
| *Psychropotes* sp. 68 | Pacific | Unavailable data | N/A | KU987536 | *Gubili et al*., *2017*; *Gebruk*, *Kremenetskaia & Rouse*, *2020* |
| *Psychropotes* sp. 69 | Northeast Atlantic | Unavailable data | N/A | KU987537 | *Gubili et al*., *2017*; *Gebruk*, *Kremenetskaia & Rouse*, *2020* |
| *Psychropotes* sp. 70 | Southern Indian | Unavailable data | N/A | KU987538 | *Gubili et al*., *2017*; *Gebruk*, *Kremenetskaia & Rouse*, *2020* |
| *Psychropotes* sp. 71 | Southern Indian | Unavailable data | N/A | KU987539 | *Gubili et al*., *2017*; *Gebruk*, *Kremenetskaia & Rouse*, *2020* |
| *Psychropotes* cf. *semperiana* | Clarion-Clipperton Zone | NHM_220 | KU519526 | N/A | *Gubili et al*., *2017* |
| *Psychropotes diutiuscauda* sp. nov. | Wallaby-Zenith Fracture Zone | IDSSE-EEB-HS175 | PP868348 | PP869371 | This study |
| *Psychropotes nigrimargaria* sp. nov. | Wallaby-Zenith Fracture Zone | IDSSE-EEB-HS176 | PP868349 | PP869372 | This study |
| *Psychropotes asperatus* sp. nov. | The Kermadec Trench | NIWA164003 | PP868347 | PP869370 | This study |
| *Psychropotes depressa* | The Kermadec Arc | NIWA164160 | PP868346 | PP869369 | This study |
| *Psychropotes depressa* | Indian Ocean: Balthazar Seamount | NMV-F308148 | PP817909 | PP778423 | *Mackenzie et al*., *2024* |
| *Psycheotrephes exigua* | Antarctica | NIWA39018 | N/A | KX874392 | *Miller et al*., *2017* |
| *Benthodytes* cf. *sanguinolenta* | Clarion-Clipperton Zone | CCZ_178 | N/A | ON400720 | *Bribiesca-Contreras et al*., *2022* |
| *Benthodytes sanguinolenta* | Antarctic | MOLAF381 | N/A | HM196507 | *O’Loughlin et al*., *2011* |
| *Benthodytes manusensis* | Manus Basin, West Pacific | MBM286091 | MH627223 | MH627222 | *Xiao*, *Li & Sha*, *2018* |
| *Benthodytes marianensis* | Mariana Trench | IDSSE-2016-0629-HS01 | MH049433 | MH049435 | *Li et al*., *2018* |
| *Benthodytes marianensis* | Clarion-Clipperton Zone | CCZ_019 | N/A | ON400682 | *Bribiesca-Contreras et al*., *2022* |
| *Benthodytes jiaolongi* | Kyushu-Palau Ridge, West Pacific | RSIO6017101 | MW992747 | MW990357 | *Yu et al*., *2022* |
| *Benthodytes sibogae* | South China Sea | MBM286090 | MZ571921 | MZ570476 | *Xiao*, *Xiao & Zeng*, *2020* |
| *Benthodytes palauta* | Kyushu-Palau Ridge | RSIO591006 | MW465752 | MW458948 | *Yu et al*., *2021* |
| *Benthodyte occidentpalauta* | Kyushu-Palau Ridge | RSIO6820405 | OP538683 | OP538553 | *Yuan*, *Wang & Zhang*, *2024* |
| *Benthodyte tetrapapillata* | Caroline Ridge | MBM286931 | MZ889521 | MZ891643 | *Xiao*, *Xiao & Zeng*, *2023* |
| *Benthodyte* sp. | Northwest Pacific Ocean | SB-1-11 | MN310402 | MN313657 | *Kremenetskaia et al*., *2021* |
| *Peniagone vignoni* | Antarctic | MOLAF721 | N/A | HM196382 | *O’Loughlin et al*., *2011* |
| *Peniagone incerta* | Antarctic | MOLN114 | N/A | HM196398 | *O’Loughlin et al*., *2011* |
| *Peniagone diaphana* | Northeast Atlantic | NOCS-DC 13369#2 | KX856725 | KX874384 | *Miller et al*., *2017* |
| *Peniagone* sp. AKM-2016 | Mexico, Gulf of California | SIO-BIC E5608 | KX856726 | KX874385 | *Miller et al*., *2017* |
| *Peniagone* sp. PE30 | Antarctic | ZMBN_PE30 | MZ563437 | MZ563440 | *Kremenetskaia et al*., *2021* |
| *Peniagone mus* | Northwest Pacific | PE1_A | MZ563459 | MZ563475 | *Kremenetskaia et al*., *2021* |
| *Peniagone coccinea* | North Atlantic | ECOMAR_B30 | MZ563446 | MZ563467 | *Kremenetskaia et al*., *2021* |
| *Peniagone purpurea* | Northwest Pacific | PE4_A | MZ563448 | MZ563469 | *Kremenetskaia et al*., *2021* |
| *Peniagone islandica* | North Atlantic | ECOMAR_H50 | MZ563444 | MZ563465 | *Kremenetskaia et al*., *2021* |
| *Scotoplanes hanseni* | Northwest Pacific | SC1_A | MZ563452 | MZ563472 | *Kremenetskaia et al*., *2021* |
| *Protelpidia murrayi* | Antarctic | MOLAF719 | N/A | HM196407 | *O’Loughlin et al., 2011* |
| *Elpidia glacialis* | Antarctic | MOLAF740 | N/A | HM196413 | *O’Loughlin et al., 2011* |
| *Elpidia kurilensis* | Northwest Pacific | EL10_A | MZ563445 | MZ563466 | *Kremenetskaia et al., 2021* |
| *Amperima robusta* | Antarctica | NIWA 37990 | KX856728 | KX874381 | *Miller et al., 2017* |
| *Pannychia* cf. *moseleyi* | USA, California | SIO-BIC E5614 | KX856732 | KX874379 | *Miller et al., 2017* |
| *Pannychia moseleyi* | USA, California | SIO-BIC E4625 | KX856731 | KX874380 | *Miller et al., 2017* |
| *Pannychia rinkaimaruae* | Japanese waters | NSMT E-13404 | N/A | LC750305 | *Ogawa et al., 2023* |
| *Pannychia nagasakimaruae* | Japanese waters | NSMT E-13401 | N/A | LC750304 | *Ogawa et al., 2023* |
| *Pannychia henrici* | Northwest Pacific Ocean | NSMT E-13464 | N/A | LC664721 | *Ogawa et al., 2022* |
| *Pannychia virgulifera* | Northwest Pacific Ocean | NSMT E-13458 | N/A | LC664750 | *Ogawa et al., 2022* |
| *Psychronaetes* sp. | Clarion-Clipperton Zone | CCZ_101 | N/A | ON400707 | *Bribiesca-Contreras et al., 2022* |
| *Psychronaetes* sp. | Clarion-Clipperton Zone | CCZ_063 | N/A | ON400690 | *Bribiesca-Contreras et al., 2022* |
| *Psychronaetes* sp. | Clarion-Clipperton Zone | CCZ_104 | N/A | ON400710 | *Bribiesca-Contreras et al., 2022* |
| *Psychronaetes* sp. | Clarion-Clipperton Zone | CCZ_103 | N/A | ON400709 | *Bribiesca-Contreras et al., 2022* |
| *Laemogone* cf. *wyvillethomsoni* | Clarion-Clipperton Zone | CCZ_062 | N/A | ON400689 | *Bribiesca-Contreras et al., 2022* |
| *Laemogone wyvillethomsoni* | Mairie Byrd Seamounts | MOLAF715 | N/A | HM196504 | *O’Loughlin et al., 2011* |
| *Laemogone* sp. | The Kermadec Trench region | NIWA164165 | N/A | PP817260 | *Unpublished data* |
| *Benthogone abstrusa* | Australia, NW Coast | NMV-MIC F146599 | KX856733 | KX856733 | *Miller et al., 2017* |
| *Benthogone rosea* | Indian Ocean: Clara Marie Seamount | NMV-F296864 | PP817917 | PP778425 | *Mackenzie et al., 2024* |
| *Enypniastes eximia* | New Zealand | SIO-BIC E6670 | KX856730 | KX874383 | *Miller et al., 2017* |
| *Enypniastes eximia* | Indian Ocean, Australia, Christmas Island | NMV F296847 | PP817912 | N/A | *Mackenzie et al., 2024* |
| *Enypniastes eximia* | Indian Ocean, Australia, Christmas Island | NMV F296837 | PP817911 | N/A | *Mackenzie et al., 2024* |
| *Pseudostichopus* sp.1 | Antarctic | SIO-BIC E6363 | KX856758 | KX874388 | *Miller et al., 2017* |
| *Pseudostichopus* sp.2 | Antarctic | SIO-BIC E5708 | KX856759 | KX874389 | *Miller et al., 2017* |

**References**

**Bribiesca-Contreras G, Dahlgren TG, Amon DJ, Cairns S, Drennan R, Durden JM, Eléaume MP, Hosie AM, Kremenetskaia A, McQuaid K, O’hara TD, Rabone M, Simon-Lledó E, Smith CR, Watling L, Wiklund H, Glover AG. 2022.** Benthic megafauna of the western Clarion-Clipperton Zone, Pacific Ocean. *ZooKeys* **1113:** 1–110 DOI 10.3897/zookeys.1113.82172.

**Gebruk AV, Kremenetskaia A, Rouse GW. 2020.** A group of species “*Psychropotes longicauda*” (Psychropotidae, Elasipodida, Holothuroidea) from the Kuril-Kamchatka Trench area (North-West Pacific). *Progress in Oceanography* **180:** 102222 DOI 10.1016/j.pocean.2019.102222.

**Gubili C, Ross E, Billett DSM, Yool A, Tsairidis C, Ruhl HA, Rogacheva A, Masson D, Tyler PA, Hauton C. 2017.** Species diversity in the cryptic abyssal holothurian *Psychropotes longicauda* (Echinodermata). Deep-Sea Research Part II: Topical Studies in Oceanography **137:** 288–296 DOI: 10.1016/j.dsr2.2016.04.003.

**O’Loughlin PM, Paulay G, Davey N, Michonneau F. 2011.** The Antarctic region as a marine biodiversity hotspot for echinoderms: Diversity and diversification of sea cucumbers. *Deep-Sea Research Part II: Topical Studies in Oceanography* **58:** 264–275 DOI 10.1016/j.dsr2.2010.10.011.

**Ogawa A, Kremenetskaia A, Hiruta SF, Shibata Y, Narimatsu Y, Miki S, Morita T, Tsuchida S, Fujiwara Y, Fujita T. 2022.** Rehabilitation of two deep-sea holothurian species in genus *Pannychia* from the northwest Pacific Ocean. *Deep-Sea Research Part II: Topical Studies in Oceanography* **202:** 105099 DOI 10.1016/j.dsr2.2022.105099.

**Ogawa A, Kobayashi I, Kohtsuka H, Fujita T. 2023.** Two new species of the bathyal holothurian genus *Pannychia* (Elasipodida, Laetmogonidae) from Japanese waters. *Zootaxa* **5323:** 105–125 DOI 10.11646/zootaxa.5323.1.6.

**Li YN, Xiao N, Zhang LP, Zhang H. 2018.** *Benthodytes marianensis*, a new species of abyssal elasipodid sea cucumbers (Elasipodida: Psychropotidae) from the Mariana Trench area. *Zootaxa* **4462(3):** 443–450 DOI 10.11646/zootaxa.4462.3.10.

**Mackenzie M, Davey N, Burghardt I, Haines ML. 2024.** A report of sea cucumbers collected on the first dedicated deep-sea biological survey of Australia’s Indian Ocean Territories around Christmas and Cocos (Keeling) Islands (Echinodermata: Holothuroidea). *Memoirs of Museum Victoria* **83:** 207–316 DOI 10.24199/j.mmv.2024.83.03.

**Miller AK, Kerr AM, Paulay G, Reich M, Wilson NG, Carvajal JI, Rouse GW. 2017.** Molecular phylogeny of extant Holothuroidea (Echinodermata). *Molecular Phylogenetics and Evolution* **111:** 110–131 DOI 10.1016/j.ympev.2017.02.014.

**Mongiardino Koch N, Tilic E, Miller AK, Stiller J, Rouse GW. 2023.** Confusion will be my epitaph: genome-scale discordance stifles phylogenetic resolution of Holothuroidea. Proceedings of the Royal Society B: *Biological Sciences* **290** DOI 10.1098/rspb.2023.0988.

**Kremenetskaia A, Gebruk A, Alt CHS, Budaeva N. 2021.** New and poorly known species of *Peniagone* (Holothuroidea, Elpidiidae) from the Northwest Pacific Ocean with Discussion on Phylogeny of the genus. *Diversity* **13(11):** 541 DOI 10.3390/d13110541.

**Xiao N, Li X, Sha Z. 2018.** Psychropotid holothurians (Echinodermata: Holothuroidea: Elasipodida) of the tropical Western Pacific collected by the KEXUE expedition with description of one new species. *Marine Biology Research* **14(8):** 816–826 DOI 10.1080/17451000.2018.1546012.

**Xiao N, Gong L, Kou Q, Li X. 2019.** *Psychropotes verrucicaudatus*, a new species of deep-sea holothurian (Echinodermata: Holothuroidea: Elasipodida: Psychropotidae) from a seamount in the South China Sea. *Bulletin of Marine Science* **95:** 421–430 DOI 10.5343/bms.2018.0041.

**Xiao Y, Xiao N, Zeng X. 2020.** New records of a genus and a species of Psychropotidae (Echinodermata: Holothuroidea: Elasipodida) from the South China Sea. *Oceanologia et Limnologia Sinica* **51(03):** 644-648.

**Xiao Y, Xiao N, Zeng X.** **2023.** *Benthodytes tetrapapillata* sp. nov., a new elasipodid sea cucumber (Elasipodida: Psychropotidae) from a seamount in the Western Pacific Ocean. *Journal of Oceanology and Limnology* **41(5):** 1978–1987 DOI 10.1007/s00343-022-2220-0.

**Yu C, Wang C, Zhang D, Zhang R. 2021.** *Benthodytes palauta*, a new species of deep-sea holothuroid (Elasipodida: Psychropotidae) from the western Pacific Ocean. *Acta Oceanologica Sinica* **40:** 50–54 DOI 10.1007/s13131-021-1937-5.

**Yu C, Zhang D, Zhang R, Wang C. 2022.** New psychropotid species (Echinodermata, Holothuroidea, Elasipodida) of the Western Pacific with phylogenetic analyses. *ZooKeys* **2022:** 99–114 DOI 10.3897/zookeys.1088.69141.

**Yuan C, Wang C, Zhang D. 2024.** *Benthodytes occidentpalauta* sp. nov., a new species of deep-sea holothuroid (Elasipodida: Psychropotidae) from the west of Kyushu-Palau Ridge in the Western Pacific Ocean. *Journal of Oceanology and Limnology* **42:** 252–262 DOI 10.1007/s00343-023-2344-x.
